# Protective Effects of Individual and Combined Low Dose Beta-Carotene and Metformin Treatments against High-Fat Diet-Induced Responses in Mice

**DOI:** 10.3390/nu13103607

**Published:** 2021-10-14

**Authors:** Bojan Stojnić, Alba Serrano, Lana Sušak, Andreu Palou, M. Luisa Bonet, Joan Ribot

**Affiliations:** 1 Grupo de Nutrigenómica, Biomarcadores y Evaluación de Riesgos, Laboratory of Molecular Biology, Nutrition and Biotechnology (LBNB), Universitat de les Illes Balears, 07122 Palma, Spain; bstojnic@gmail.com (B.S.); albaserranobengoechea@gmail.com (A.S.); lana.susak.17@gmail.com (L.S.); andreu.palou@uib.es (A.P.); joan.ribot@uib.es (J.R.); 2 Institut d’Investigació Sanitària Illes Balears (IdISBa), 07120 Palma, Spain; 3 CIBER de Fisiopatología de la Obesidad y Nutrición (CIBERobn), 07122 Palma, Spain

**Keywords:** phytochemical, carotenoids, cotherapy, obesity

## Abstract

Anti-obesity activity has been reported for beta-carotene (BC) supplementation at high doses and metformin (MET). We studied whether BC treatment at a closer to dietary dose and MET treatment at a lower than therapeutic dose are effective in ameliorating unwanted effects of an obesogenic diet and whether their combination is advantageous. Obesity-prone mice were challenged with a high-fat diet (HFD, 45% energy as fat) for 4 weeks while receiving a placebo or being treated orally with BC (3 mg/kg/day), MET (100 mg/kg/day), or their combination (BC+MET); a fifth group received a placebo and was kept on a normal-fat diet (10% energy as fat). HFD-induced increases in body weight gain and inguinal white adipose tissue (WAT) adipocyte size were attenuated maximally or selectively in the BC+MET group, in which a redistribution towards smaller adipocytes was noted. Cumulative energy intake was unaffected, yet results suggested increased systemic energy expenditure and brown adipose tissue activation in the treated groups. Unwanted effects of HFD on glucose control and insulin sensitivity were attenuated in the treated groups, especially BC and BC+MET, in which hepatic lipid content was also decreased. Transcriptional analyses suggested effects on skeletal muscle and WAT metabolism could contribute to better responses to the HFD, especially in the MET and BC+MET groups. The results support the benefits of the BC+MET cotreatment.

## 1. Introduction

The consumption of unbalanced diets with excess fat and/or sucrose promotes obesity and associated comorbidities such as insulin resistance, dyslipidemia, hypertension and fatty liver. Pharmacological treatments available against these health risks/diseased states entail problems such as low adherence to chronic drug prescription, economic burden or side effects [1]. In parallel, many dietary compounds, especially phytochemicals, are being recognized as having interesting properties for the management of obesity and associated metabolic complications, owing to their ability to impact key biological target molecules and processes [1]. The combination of pharmaceuticals with dietary bioactive compounds emerges in this context as a potential strategy to simultaneously tackling the same or different health/therapeutic targets in complementary, cumulative or synergistic manners, which may result in more effective treatments, decreased drug doses needed or improved patient’s response to treatment [1].

Beta-carotene (BC) is a carotenoid phytochemical that, in mammals, can function as a precursor of the vitamin A retinoids (i.e., retinol, retinal, retinoic acid), as well as having vitamin A-independent activities [2]. Dietary carotenoids, including BC and subcutaneously administered retinoids such as retinoic acid, ameliorate obesity and obesity development in animal models, and there is evidence of the same sense in humans [3,4,5,6,7]. Additionally, the beneficial effects of BC administration on blood glucose levels [8] and BC conversion into vitamin A on blood lipids and atherosclerosis progression [9,10] were described in pre-clinical studies.

The anti-adiposity effect of supplemental BC in mice appears to be dependent on BC conversion to retinoids and involves the inhibition of a master pro adipogenic/lipogenic transcription factor, peroxisome proliferator-activated receptor gamma (PPARγ), in adipose cells and fat depots [11,12]. Another determinant of a lean phenotype is a high rate of substrate oxidation, which in adipose tissues is often linked to enhanced uncoupling protein 1 (UCP1)-mediated thermogenesis in activated brown adipose (BAT) and “browned” white adipose tissue (WAT). BAT activation and WAT browning are promoted in mice following subcutaneous administration of all-trans retinoic acid [13,14,15], a treatment that also promotes substrate (mainly fatty acid) catabolism in non-adipose tissues such as skeletal muscle and the liver [16,17]. The impact of dietary BC supplementation on thermogenesis in adipose tissues remains less clear, and it has been studied mainly in ferrets, where it greatly depends on the BC formulation used [18,19].

Metformin (MET), a biguanide derivative, is best known as a first-line treatment drug for type 2 diabetes owing to its well-documented blood glucose-lowering effects and safety records [20]. Doses clinically used for this purpose range from 1000 to 2500 mg/day, where 2000 mg/day may represent the optimal dose for most patients [21]. MET is receiving increasing attention in recent years as a potential anti-obesity drug on the basis of clinical and animal studies [22,23,24,25]. MET-induced bodyweight loss is mainly attributed to decreased caloric intake, resulting from direct and indirect (gut–brain axis mediated) effects of the drug on central circuitries controlling appetite [25]. Additionally, changes in the microbiome [25] and increases in systemic energy expenditure and metabolism were described in rodents and humans after MET treatment that may contribute to MET-induced body weight or fat loss [26,27].

In previous studies in rodents, anti-adiposity and glucose-lowering activities of BC supplementation were demonstrated at high BC doses of ~35 mg/kg/day, corresponding to ~170 mg/day in a 60-kg human [28], i.e., about 38-fold the average daily BC intake through the diet in European countries (which is ~3 to 6 mg/day) [2]. Here, we aimed to investigate if BC treatment at a more physiological (closer to dietary) dose and MET treatment at a lower than a therapeutic dose are effective in ameliorating the development of diet-induced obesity and whether their combination can counteract unwanted effects of an obesogenic diet more effectively than the individual treatments alone. To this end, we treated mice on a high-fat diet with 3 mg BC/kg mice/day (corresponding to ~14 mg/day for a 60-kg human), 100 mg MET/kg mice/day (corresponding to ~500 mg/day for a 60-kg human), or their combination.

## 2. Materials and Methods

### 2.1. Animal Experiment

The experiment was carried out following the protocols reviewed and approved by the Bioethical Committee of the University of the Balearic Islands (Resolution number CEEA 43/07/15). Guidelines of the University for the correct use, accommodation and care of laboratory animals were followed. Animals (3 per cage) were housed under standard conditions of controlled temperature (22 °C), a 12-h light–dark cycle (light on from 8:00 to 20:00 h) and free access to food and tap water.

Forty-five 7-week-old C57BL/6J male mice (Jackson Laboratory, ME, USA) that were pre-habituated to a defined normal-fat diet (NFD, 10% energy as fat) for one week were used. The animals were given daily, during one-week placebo (i.e., a mix of water and olive oil, used as vehicle), BC (3 mg/kg/day), MET (100 mg/kg/day), or the combination of BC (3 mg/kg/day) plus MET (100 mg/kg/day) while still on the NFD, and then challenged with a defined high-fat diet (HFD, 45% energy as fat) for 4 weeks while continuing receiving the same daily supplements, making out the HF control group and the BC, MET and BC+MET groups (9 animals/group). A fifth group run in parallel was given the vehicle and remained on the NFD during the entire experimental period (NF control group). The defined diets were obtained from Research Diets (New Brunswick, NJ, USA; NFD D12450J and HFD D12451); BC all-trans from Sigma-Aldrich (Madrid, Spain) and MET hydrochloride from Acofarma (Madrid, Spain). BC was dissolved in olive oil, and MET was dissolved in water. Treatments or vehicles (20 μL) were administered orally, through a pipette, roughly at the same time of the day (between 10:00 and 11:00 h), ensuring complete ingestion of the treatment.

Body composition was analyzed immediately before the HFD/NFD challenge, using an Echo MRI-700 body composition analyzer (Echo Medical Systems LLC, Houston, TX, USA). Bodyweight and food intake were recorded twice a week from the beginning of the treatment until sacrifice. Bodyweight gain was calculated as the difference of body weight from the initial (at the beginning of the treatment or HFD/NFD challenge) to the final weight for each specific time-point and expressed as grams. Cumulative energy intake was estimated on a per-cage basis from the amount of food consumed and its caloric equivalence and expressed in relation to body weight (as kJ/g body weight). On the 4th week of the HFD/NFD challenge, body composition analyses were repeated on all animals, internal body temperature was measured—by gently inserting the probe of a calibrated thermometer (RS 612–849; RS Components, Madrid, Spain) in the rectum, with Vaseline used as lubricant, and the animals were submitted to a 6 h-fast (from 06:00 to 12:00) after which tail blood was taken for the assessment of insulin resistance and sensitivity (see Section 2.2).

Animals were euthanized by decapitation, under fed conditions, after 30 days of dietary challenge within the first 2 h of the light cycle. Blood collected from the neck was used to prepare serum, which was stored at −20 °C. Interscapular BAT, inguinal WAT (iWAT), epididymal WAT (eWAT), retroperitoneal WAT (rWAT), gastrocnemius skeletal muscle and liver were dissected entirely, weighed, snap-frozen in liquid nitrogen and stored at −80 °C until processed. Samples of iWAT and BAT were fixed for subsequent histological studies (see Section 2.6). Adiposity index was defined as the sum of the mass of all WAT depots dissected expressed as a percentage of body weight, and visceral/subcutaneous ratio as the ratio between iWAT mass and the sum of the mass of eWAT and rWAT.

### 2.2. Blood Parameters and Surrogate Indexes of Insulin Resistance and Sensitivity

Blood glucose was determined using an Accu-Chek Aviva system (Roche Diagnostics, Risch, Switzerland). Commercial kits for measurement of serum insulin (Mercodia, Uppsala, Sweden) and non-esterified fatty acids (NEFA; Wako Chemicals GmbH, Neuss, Germany) were applied following the manufacture’s protocols. Indexes of insulin resistance and sensitivity were derived from glucose, insulin and NEFA levels in plasma prepared from the blood of 6 h-fasted animals. The homeostatic model assessment for insulin resistance (HOMA-IR) score was calculated as HOMA-IR = [insulin (μU/L) × glucose (mmol/L)/22.5] [29], and the revised quantitative insulin sensitivity check index (R-QUICKI) as R-QUICKI = 1/[log glucose (mg/dL) + log insulin (μU/mL) + log NEFA (mM/L)] [30].

### 2.3. Liver Total Lipid Content

Total hepatic lipids were extracted from 50 to 100 mg of tissue as previously described [31]. Lipid content per gram of tissue was determined from the weight of the tubes following evaporation of the final hexane extract, subtracting the initial (clean) tube weight and considering the initial amount of tissue used.

### 2.4. RNA Isolation, Retrotranscription and Real-Time PCR Amplification

Total RNA was extracted from tissue samples using commercial E.Z.N.A. Total RNA kit I (Omega Bio-Tek, Norcross, GA, USA), following the supplier’s instructions. Isolated RNA was quantified using a Nanodrop ND 1000 spectrophotometer (Nano-Drop Technologies Inc., Wilmington, NC, USA), and its integrity was confirmed by agarose gel electrophoresis. Reverse transcription, PCR amplification of selected cDNAs and data analysis were as previously described [32]. The sequences of the primers used (obtained from Sigma-Aldrich) are available upon request. Data were normalized against beta-actin as the reference.

### 2.5. Immunoblotting

Immunoblotting was used to semi-quantify UCP1, Mitofusin 2 (MFN2) and PPARα in BAT. For this, tissue was homogenized 1:15 (*w*:*v*) at 4 °C in RIPA Lysis Buffer 1× (0.1% SDS, 0.5% sodium deoxycholate, 1% Nonidet P-40, 150 mmol/L NaCl and 50 mmol/L Tris-HCl, pH 8.0), with protease and phosphatase inhibitor cocktail 1× (Thermo Fisher Scientific, Rockford, IL, USA). The homogenate was centrifuged at 7500 *g* for 5 min at 4 °C, and the supernatant was used for protein analyses. Total protein content was measured with the Pierce BCA protein assay kit (Thermo Fisher Scientific). A total of 19 μg of protein from BAT was solubilized with 3:1 (*v*/*v*) of Laemmli Sample Buffer (Bio-Rad, Hercules, CA, USA) containing 1% 2-β-mercaptoethanol (Sigma-Aldrich), boiled for 3–4 min, fractionated in a 12% precast Criterion TGX polyacrylamide gel (Bio-Rad) and transferred onto a 0.2 μm nitrocellulose membrane using a Trans-blot Turbo semi-dry transfer apparatus (Bio-Rad). After blocking with Odyssey Blocking Buffer (Li-COR Biosciences, Lincoln, NE, USA), the membranes were incubated overnight at room temperature with primary antibodies (1:1000 in Tris Buffered Saline Tween 20, TBS-T) against β-actin (#3700, Cell Signalling, Danvers, MA, USA) used as reference protein; UCP1 (GTX10983, GeneTex, Irvine, CA, USA); MFN2 (HPA030554, Sigma-Aldrich); and PPARα (ab8934, Abcam, Cambridge, UK). The membranes were then incubated with corresponding secondary IRDye antibodies (1:10000 in TBS-T, 1 h at room temperature). Membranes were successively probed for the target proteins, with a stripping step with Stripping Buffer 5× (LI-COR Biosciences) before each reprobing process. Protein bands were detected by infrared fluorescence and quantified using an Odyssey near-infrared fluorescence scanner (Li-COR Biosciences). The signals of every protein of interest were normalized to the signal of β-actin.

### 2.6. Histology and Immunohistochemistry

Tissue samples were fixed by immersion in 4% paraformaldehyde in 0.1 M sodium phosphate buffer, pH 7.4, overnight at 4 °C, dehydrated in a graded series of ethanol, cleared in xylene and embedded in paraffin blocks for light microscopy. Five-micrometer-thick sections of tissues were cut with a microtome, mounted on slides and stained with hematoxylin/eosin. Morphometric analysis of inguinal WAT sections was performed by the digital acquisition of adipose tissue areas using AxioVision 40V 4.6.3.0 software and a Zeiss Axioskop 2 microscope equipped with an AxioCam ICc3 digital camera (Carl Zeiss S.A., Barcelona, Spain). Distributions of adipocyte size were obtained from individual data of cell sizes. Immunohistochemical detection of UCP1 in BAT sections was performed essentially as previously described [32], using a polyclonal antibody against UCP1 (Catalog number GTX112784, GeneTex, Irvine, CA, USA) as the primary antibody.

### 2.7. Statistical Analysis

Data are expressed as mean ± SEM. Statistical significance of differences between groups was assessed by one-way ANOVA, followed by Tukey’s Honest Significant Difference post hoc test. When indicated in the text, Dunnett’s multiple comparison post hoc test was used to identify significant differences against a selected control group, or two-way ANOVA was used to compare the β-carotene and metformin effects in HFD-fed animals. Analysis was carried out with IBM SPSS Statistics for Windows, Version 27.0. (IMB Corp., New York, NY, USA) and Microsoft Excel (Microsoft, Redmond, WA, USA). The threshold of significance was set at *p* < 0.05.

## 3. Results

### 3.1. Biometric and Adiposity Parameters

In our experimental design, obesity-prone mice were given daily for one week the vehicle (HF control group), BC, MET, or BC+MET under NFD feeding conditions, and then challenged with an obesogenic HFD for 4 weeks while continuing receiving same supplements. The fifth group of animals given the vehicle and fed the NFD throughout the entire experimental period (NF group) served as a control for the HFD effects. The one-week pre-treatments had no impact on the animals’ body weight and composition so that there were no significant differences in body weight, fat body mass and lean body mass percentage (as assessed by ECO-MRI) between the experimental groups at the start of the HFD feeding period (not shown).

**Figure 1:**
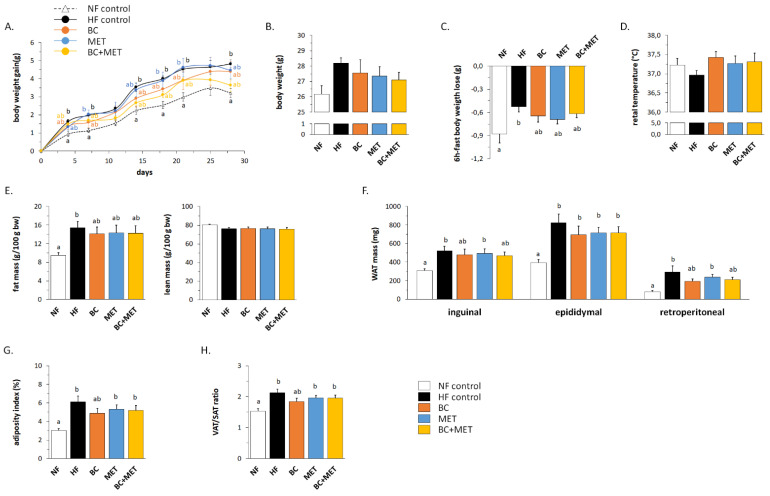
Evolution of body weight (bw) gain during the dietary challenge (**A**), body weight at the end of experiment (**B**), body weight lost upon a 6 h-fast (**C**), rectal temperature (**D**) and body composition (**E**) 5 to 7 days prior the sacrifice. Inguinal, epididymal and retroperitoneal white adipose tissue (iWAT, eWAT and rWAT) weights (**F**), adiposity index as the sum of WAT weights as percent body weight (**G**) and the visceral (VAT: eWAT and rWAT) to subcutaneous (SAT: iWAT) ratio (**H**) at the end of the experiment. Obesity-prone mice were challenged with a high-fat diet for 4 weeks while receiving placebo (HF control) or being treated orally with BC (3 mg/kg/day), MET (100 mg/kg/day), or their combination (BC+MET); a fifth group received placebo and was kept on a normal-fat diet (NF control). Data are means ±SEM of seven to nine animals per group. To compare between groups, one-way ANOVA followed by Tukey’s Honest Significant Difference post hoc test was used: bars not sharing a common letter (a and b) are significantly different (a ≠ b) (*p* < 0.05).

The evolution of body weight gain during the HFD/NFD challenge is shown in Figure 1A. The HF control group displayed greater body weight gain than the NF group from day 4 of HFD feeding onwards, as expected. Similar results were found for the MET treated mice, whereas body weight gain was somewhat attenuated in the BC treated mice, and especially, the BC+MET mice. Accordingly, Dunnett’s test of data of the last day of the follow-up indicated a significantly increased body weight gain vs. that of the NF group in the HF (*p* = 0.008) and the MET (*p* = 0.040) groups, but not in the BC (*p* = 0.051) and the BC+MET (*p* = 0.461) groups. Differences in body weight gain were not attributable to differences in energy intake, which was similar in all experimental groups throughout the whole dietary challenge (data not shown). At the end of the experiment, body weight was significantly increased over that of NF mice only in the untreated HF controls (*p* = 0.034, Dunnett’s test) (Figure 1B).

Bodyweight loss upon a 6-h fast and rectal temperature—both measured in the fourth week of the dietary challenge—were used as surrogate indicators of energy expenditure. HFD feeding was associated with a decrease in body weight loss upon fasting that reached statistical significance only in the untreated HF control group (Figure 1C). In good concordance, rectal temperature was minimal in the HF control group (Figure 1D). These results are suggestive of a decreased energy expenditure on the HFD that likely contributes to body weight gain on this diet and is counteracted in part by BC, MET and BC+MET treatments.

Body composition analyses performed in the live animals by the end of the dietary challenge evidenced an HFD-induced increase in body fat mass (expressed as a percentage of body weight) that was attenuated in the three HFD-fed treated groups (Figure 1E). Data at sacrifice showed HFD-induced increases in inguinal and retroperitoneal fat depot mass to be attenuated in the BC and the BC+MET groups (Figure 1F). In the BC group, attenuation of HFD-induced increases in the adiposity index and the visceral to subcutaneous (VAT/SAT) adipose tissue ratio were also evidenced (Figure 1G,H). No differences were observed among groups in the relative weights of BAT and liver (data not shown).

**Figure 2:**
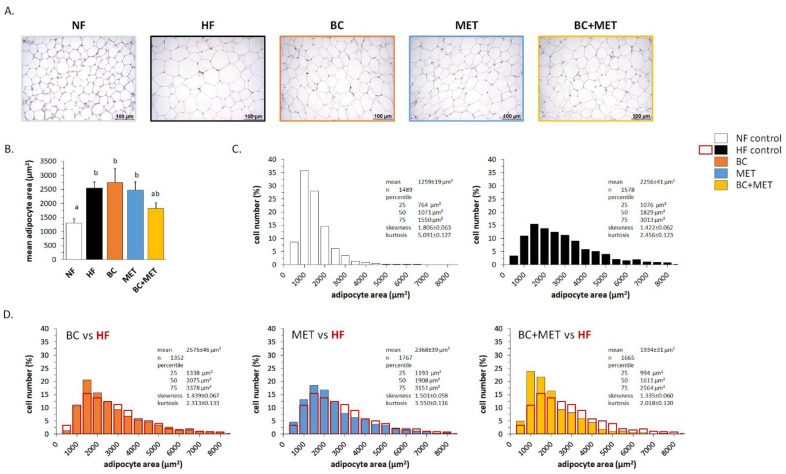
Representative microphotographs illustrating adipocyte size (**A**), mean adipocyte area (**B**) and distribution of adipocytes size (**C**) in iWAT at the end of the experiment. Obesity-prone mice were challenged with a high-fat diet for 4 weeks while receiving placebo (HF control) or being treated orally with BC (3 mg/kg/day), MET (100 mg/kg/day), or their combination (BC+MET); a fifth group received placebo and was kept on a normal-fat diet (NF control). Data are means ±SEM of five to seven animals per group. In B, to compare between groups, one-way ANOVA followed by Tukey’s Honest Significant Difference post hoc test was used: bars not sharing a common letter (a and b) are significantly different (a ≠ b) (*p* < 0.05). In (**C**,**D**), between 200 and 300 cells per animal were included in the analysis of the distribution of adipocytes size. The area of individual adipocytes was measured using a quantitative morphometric method at 20× magnification with the assistance of Axio Vision software. Adipocyte size distribution was statistically different with *p* < 0.001 between the NF control and all HF groups, and between the HF control and HF treated groups, except for the MET group were *p* = 0.029, according to the Kolmogorov–Smirnov test. In (**D**), the distribution of adipocytes size of HF control group is shown overlapping the one of each HF treated group.

Microscopical examination and morphometric analysis of the inguinal fat depot (iWAT) revealed a significant increase in mean adipocyte area following HFD feeding in the HF, BC and MET groups, but not in the BC+MET group (Figure 2A,B). Detailed analysis revealed a decrease in the percentage of small adipocytes and an increase in the percentage of large adipocytes in the HF group as compared to the NF group (Figure 2C), thus reflecting HFD-induced adipocyte hypertrophy. Similar HFD-induced changes were observed in the BC mice and the MET mice, whose iWAT adipocyte size distribution was similar to that of HF mice (Figure 2D). In contrast, compared with the HF control mice, BC+MET mice had after HFD an increased percentage of small adipocytes and a decreased percentage of large adipocytes in iWAT (Figure 2D), suggesting increased hyperplasic and decreased hypertrophic tissue expansion in the BC+MET mice. No signs of WAT browning—such as the emergence of brown-like adipose cells with a multilocular intracellular lipid distribution—were noticed in any of the experimental groups.

### 3.2. Glucose Control-Related Parameters

**Figure 3:**
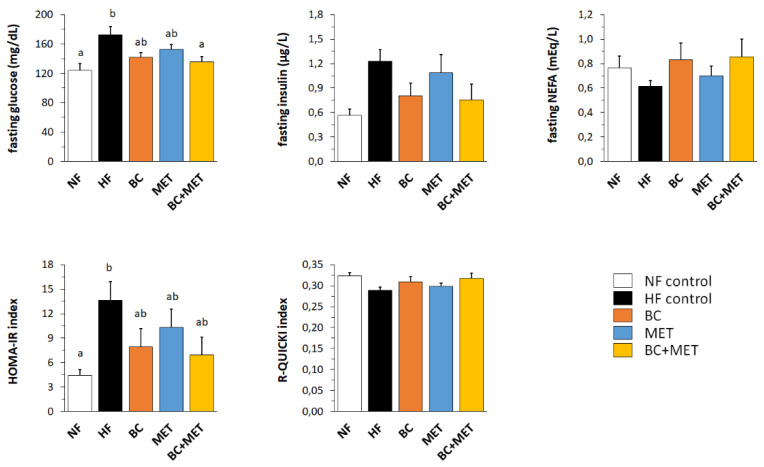
Blood glucose, serum insulin, serum NEFA and insulin resistance/sensitivity indexes (HOMA-IR and R-QUICKI) 5 to 7 days prior to the sacrifice and after a 6-h fast. Obesity-prone mice were challenged with a high-fat diet for 4 weeks while receiving placebo (HF control) or being treated orally with BC (3 mg/kg/day), MET (100 mg/kg/day), or their combination (BC+MET); a fifth group received placebo and was kept on a normal-fat diet (NF control). Data are means ± SEM of seven to nine animals per group. To compare between groups, one-way ANOVA followed by Tukey’s Honest Significant Difference post hoc test was used: bars not sharing a common letter (a and b) are significantly different (a ≠ b) (*p* < 0.05).

Parameters related to glucose control and insulin sensitivity were assessed after a 6-h fast on the fourth week of the HFD/NFD challenge (Figure 3). HFD feeding resulted, as expected, in increased fasting blood glucose, insulin (*p* = 0.020, Dunnett’s test) and HOMA-IR index in the HF control group as compared to the NF group, indicating impaired glucose control and decreased insulin sensitivity. These effects were attenuated in the treated groups, and especially in the BC and BC+MET groups, as indicated by the results of Dunnett’s tests. Further, two-way ANOVA analysis of the HFD-fed groups indicated a significant or nearly significant effect of BC lowering fasting blood glucose (*p* = 0.004), insulin (*p* = 0.047) and HOMA-IR (*p* = 0.053), and no significant effect of MET on these parameters (*p* = 0.106, *p* = 0.610 and *p* = 0.351, respectively). The glucose-lowering effect was most robust in the BC+MET group (Figure 3).

### 3.3. Energy Metabolism-Related Gene Expression in Brown Adipose Tissue

**Figure 4:**
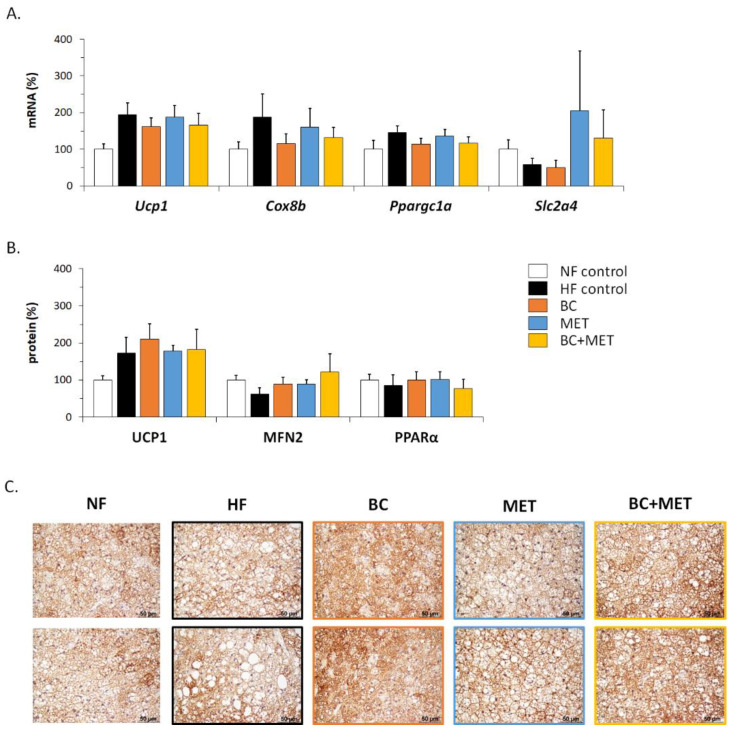
mRNA levels (**A**) and protein levels (**B**) of the indicated genes in BAT, and representative microphotographs of two mice per group illustrating BAT activation and uncoupling protein 1 (UCP1) immunostaining (**C**) at the end of the experiment. Obesity-prone mice were challenged with a high-fat diet for 4 weeks while receiving placebo (HF control) or being treated orally with BC (3 mg/kg/day), MET (100 mg/kg/day), or their combination (BC+MET); a fifth group received placebo and was kept on a normal-fat diet (NF control). Data are means ± SEM of five to nine animals per group and are expressed relative to the mean value of the NF control group, which was set to 100.

There were no significant differences among groups according to ANOVA analysis regarding the expression in BAT of UCP1—the key molecular effector of thermogenesis—and other thermogenesis and fuel metabolism-related genes assayed at the mRNA or protein level, including *Cox8b* mRNA, *Ppargc1a* mRNA, *Slc2a4* mRNA, PPARalpha protein and mitofusin-2 (MFN2) protein (Figure 4A,B). Nevertheless, several results can be highlighted. The lowest BAT UCP1 mRNA and protein levels were found in the NF control group, consistent with the well-known effect of HFD feeding to induce UCP1 expression in BAT [33]. Maximal levels of *Slc2a4* mRNA—encoding facilitated glucose transporter 4 (GLUT4) —were found in BAT of groups receiving MET, either alone or in combination with BC, yet with high interindividual variability (Figure 4A). Levels of MFN2 in BAT were higher in the HFD-fed treated groups than in the untreated HF control group (Figure 4B). Increased glucose uptake [34] and MFN2 function [35] are both keys to BAT oxidative metabolism and thermogenic activity and, therefore, observed trends are suggestive of increased BAT metabolism in the treated HFD-fed groups. Interestingly, microscopical examination of BAT sections immunostained for UCP1 revealed a more active BAT appearance (i.e., smaller lipid droplets) together with a greater UCP1 positivity in the treated HFD-fed groups, especially those receiving BC, compared with the HF control group (Figure 4C).

### 3.4. Energy Metabolism-Related Gene Expression in White Adipose Tissue Depots

**Figure 5:**
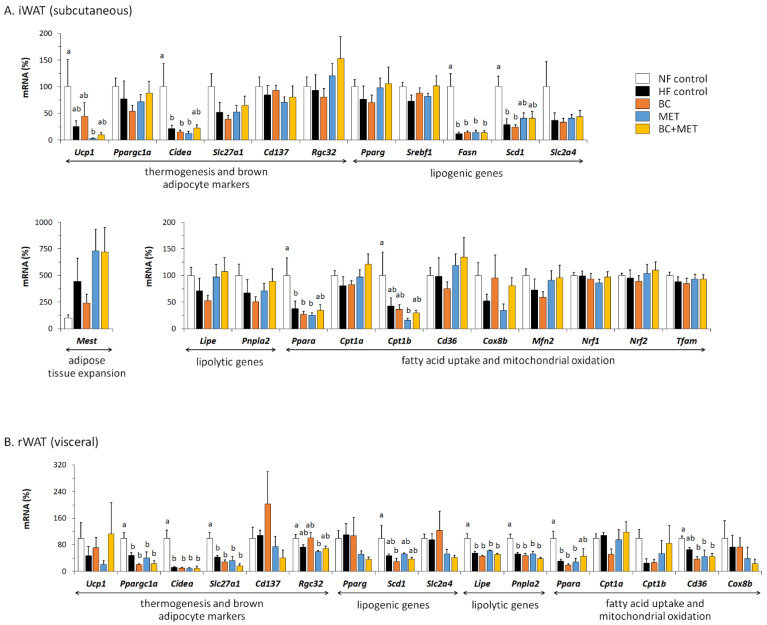
mRNA levels of the indicated genes in subcutaneous iWAT (**A**) and visceral rWAT (**B**) at the end of the experiment. Obesity-prone mice were challenged with a high-fat diet for 4 weeks while receiving placebo (HF control) or being treated orally with BC (3 mg/kg/day), MET (100 mg/kg/day), or their combination (BC+MET); a fifth group received placebo and was kept on a normal-fat diet (NF control). Data are means ± SEM of five to nine animals per group and are expressed relative to the mean value of the NF control group, which was set to 100. To compare between groups, one-way ANOVA followed by Tukey’s Honest Significant Difference post hoc test was used: bars not sharing a common letter (a and b) are significantly different (a ≠ b) (*p* < 0.05).

The results of gene expression analyses performed on subcutaneous (inguinal) and visceral (retroperitoneal) WAT are shown in Figure 5A,B, respectively. HFD feeding associated with a number of transcriptional responses in iWAT that were apparent and statistically significant in all HFD-fed groups regardless of treatments as compared with the NF group, namely: (i) a downregulated expression of genes related to thermogenesis, mitochondrial oxidative metabolism and brown adipocyte markers—*Ucp1*, *Cidea, Ppara* and *Cpt1b*; (ii) a downregulated expression of the lipogenic genes *Fasn* and *Scd1,* involved in the de novo synthesis of fatty acids. Other HFD-induced transcriptional responses recognizable (though statistically non-significant) in all HFD-fed groups were a downregulated expression of *Slc2a4,* the GLUT4 gene, which can also be viewed as a lipogenic gene in tissues capable of de novo lipogenesis, and an upregulated expression of *Mest,* which is considered a marker of adipose tissue expansion [36]. Trends to HFD-induced decreases in the expression of *Ppargc1a* (encoding PGC1ɑ, a key transcriptional coactivator for cellular oxidative metabolism), *Mfn2* (encoding MFN2, related to the transfer of fatty acids to the mitochondria for oxidation [35,37]) and lipolytic genes *Lipe* and *Pnpla2* (encoding hormone-sensitive lipase and adipose triglyceride lipase, respectively) were present in the HF control group, more marked in the BC group, yet lacking in the BC+MET group. Decreased expression of *Mfn2* [37] and of lipolytic genes [38] in WAT following HFD feeding was previously described.

In the rWAT (visceral) depot, a number of transcriptional responses to HFD feeding regardless of treatments were also observed that are in line with expected changes, in particular a downregulated expression of genes related to browning/oxidative metabolism (*Ppargc1a, Cidea*, *Slc27a1, Ppara, Cd36*), fatty acid synthesis (*Scd1*) and lipolysis (*Lipe*, *Pnpla2*) (Figure 5B). HFD-induced downregulation of *Ppara* expression in rWAT was attenuated in the BC+MET group. *Ppara* encodes a PPAR isoform whose transcriptional activity favors fatty acid oxidation in cells. Furthermore, among the HFD-fed groups, BC+MET and MET groups had the highest expression levels in rWAT of another oxidative metabolism-related gene, *Cpt1b* (encoding a key enzyme for mitochondrial long-chain fatty acid oxidation), and the lowest of lipogenic genes *Pparg* (encoding the master transcription factor for adipogenesis/ lipogenesis, PPARγ) and *Slc2a4* (encoding GLUT4), although for these three genes differences between groups failed to reach statistical significance.

### 3.5. Liver Parameters

**Figure 6:**
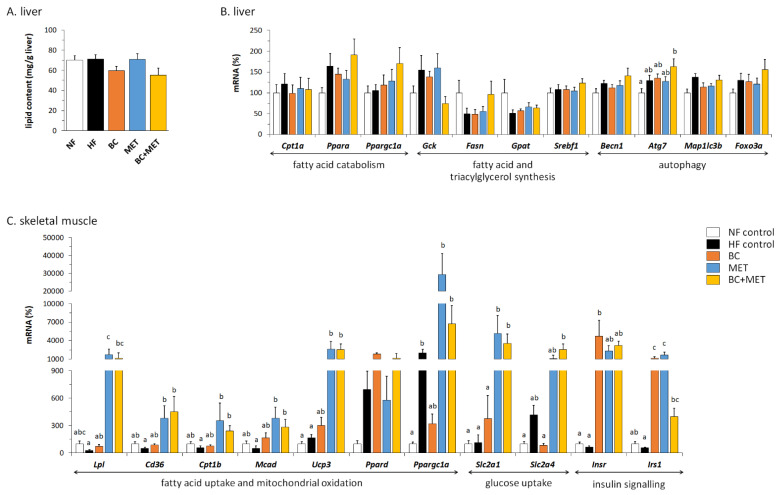
Liver total lipid content (**A**), mRNA levels of the indicated genes in liver (**B**) and skeletal muscle (**C**) at the end of the experiment. Obesity-prone mice were challenged with a high-fat diet for 4 weeks while receiving placebo (HF control) or being treated orally with BC (3 mg/kg/day), MET (100 mg/kg/day), or their combination (BC+MET); a fifth group received placebo and was kept on a normal-fat diet (NF control). Data are means ± SEM of five to nine animals per group, and mRNA levels are expressed relative to the mean value of the NF control group, which was set to 100. To compare between groups, one-way ANOVA followed by Tukey’s Honest Significant Difference post hoc test was used.

Liver total lipid content was not affected by the dietary HFD challenge imposed (Figure 6A), possibly owing to its relatively short duration (4 weeks) and mildness (45% energy as fat). However, there was a trend towards decreased hepatic lipid content in the BC mice and the BC+MET mice compared with the HF control and the MET mice. Two-way ANOVA analysis of the HFD-fed groups indicated a statistically significant (*p* = 0.02) effect of BC (but not of MET) lowering liver lipid content.

In order to investigate possible mechanisms underlying changes in total liver lipid content, hepatic expression levels of genes related to fatty acid catabolism (*Cpt1a*, *Ppara*, *Ppargc1a*), fatty acid and triacylglycerol synthesis (*Gck, Fasn*, *Gpat*, *Srebf1*) and autophagy (*Becn1*, *Atg7, Map1lc3b*, *Foxo3a*) were analyzed (Figure 6B). The latter was included because autophagy of lipid droplets (lipophagy) is increasingly recognized to contribute to decreased hepatic steatosis [39]. HFD feeding induced an upregulation of the hepatic expression of *Atg7* that was maximal and statistically significant in the BC+MET group. Similar changes, though statistically non-significant, were present for other autophagy-related genes, namely *Becn1* and *Foxo3a*. It is also noteworthy that the BC+MET group had the highest expression levels of *Ppara* and *Ppargc1a* and the lowest of the glucokinase gene (*Gck*) among the HFD-fed groups.

### 3.6. Gene Expression in Skeletal Muscle

Expression levels of selected genes in skeletal muscle are shown in Figure 6C. Compared with NF and HF controls, MET and BC+MET mice had highly increased muscle expression levels of genes for proteins involved in fatty acid extraction from triglycerides of circulating lipoproteins (*Lpl*), cellular and mitochondrial uptake of fatty acids (*Cd36*), mitochondrial fatty acid oxidation (*Cpt1b*, *Mcad, Ucp3*) and basal glucose uptake (*Slc2a1*). Expression levels of these genes were, in general, comparable in the muscle of MET and BC+MET mice. BC treatment alone had less effect on the expression of the aforementioned genes, although it promoted *Ucp3* expression and counteracted trends to downregulation of *Lpl*, *Cd36*, *Cpt1b* and *Mcad* observed in the muscle of HF control mice. Muscle expression levels of *Ppargc1a* and *Slc2a4* were induced or trended so in the HF control group, and induction of these genes was counteracted in the BC group and potentiated in the groups receiving MET, alone or combined with BC. *Ppard* expression tended to be increased in all HFD-fed groups and most consistently in the BC group. Interestingly, both BC and MET treatments induced the expression in muscle of genes for proteins in the insulin signaling pathway (*Insr* and *Irs1*), yet without additive effects in the combined BC+MET treatment group: on the contrary, *Irs1* expression was less induced in the BC+MET group than in the BC and MET groups.

## 4. Discussion

The combination of pharma drugs with dietary bioactive compounds is a potential strategy in obesity management. Previous works demonstrated anti-obesity effects of BC supplementation in animal models yet at very high doses. Here, we show that, in a mouse strain prone to obesity, treatments with BC at a lower, closer to dietary dose, MET at a lower than the therapeutic dose, and their combination can partially oppose HFD-induced body weight and body fat accumulation, likely by promoting energy expenditure. Treatment with BC, alone or in cotreatment with MET, more than MET alone, provided additional benefits favoring glucose control and insulin sensitivity on an HFD and decreasing liver lipid content. Interestingly, the BC+MET cotreatment was most effective in counteracting HFD-induced body weight gain and hyperglycemia, and, importantly, it distinctly favored hyperplasic over hypertrophic subcutaneous WAT expansion upon HFD feeding. This preponderance of the hyperplasic component of obesity is of interest since it was described to correlate with a healthier adipose tissue expansion. In particular, adipocyte hyperplasia (entailing recruitment and differentiation of adipose precursor cells), as opposed to hypertrophy (entailing adipocyte enlargement) in subcutaneous adipose tissue, appears to be protective from obesity-associated metabolic complications such as insulin resistance [40,41].

Development of HFD-induced obesity in our C57Bl/6J mice was independent of changes in energy intake, in accordance with previous studies using this model [42], and was linked to signs of decreased energy expenditure that were most evident in the untreated HF control group, such as decreased body weight lost upon a short-term fast. At the molecular level, this phenotype is associated with a decreased expression of genes related to thermogenesis, mitochondrial oxidative metabolism and brown adipocyte markers in the subcutaneous iWAT depot, in keeping with the concept that, in times of dietary fat surplus, brown/beige adipocytes in iWAT are displaced by white adipocytes, in order to store fat efficiently. Indeed, downregulation of *Ucp1* expression (and of mitochondria biogenesis) in iWAT after HFD feeding, as observed in this work, was consistently reported in mice (revised in [33]). To be noted, HFD-induced downregulation of *Ucp1* is typically seen in the iWAT depot, which is a depot otherwise prone to browning [43] and not so consistently in the visceral WAT depots, and takes place in parallel with HFD-induced upregulation of *Ucp1* in BAT [33]; results herein agree with this picture. Likewise, downregulation of genes for proteins related to fatty acid catabolism and mitochondrial substrate oxidation in WAT depots after short and long-term HFD feeding was reported [38,44], as well as other HFD-induced transcriptional responses observed here in WAT depots, such as upregulation of *Mest*—a marker of adipose tissue expansion [36]—and a downregulation of genes for proteins involved in fatty acid synthesis [38,44].

The results of body weight loss upon a short-term fast and rectal temperature suggested that treatments effects opposing HFD-induced body weight and fat mass accumulation were attributable to the promotion of energy expenditure. This promotion could contribute to an enhanced BAT metabolism, as suggested by microscopical examination of UCP1-immunostained BAT sections, showing brown adipocytes less fatty and more densely stained in the treated HFD-fed groups, especially those receiving BC compared with the HF control group. This is, to our knowledge, the first study to show signs of an upregulatory effect of oral BC supplementation on BAT activation in mice, even though effects in this sense are well-known for vitamin A treatment in the form of retinoic acid [13,14]. As for MET treatment, the early literature showed a lack of effect on BAT activity in obese rats [45] and normal mice [46], yet more recent studies indicate that MET is uptaken by BAT in vivo in mice [47] and can enhance BAT mitochondrial content and thermogenic capacity [48,49,50]. Our results indicate that the effects of single BC and MET treatments favoring activation of BAT in mice on an HFD are not additive.

Besides effects on BAT, effects on WAT metabolism could contribute to the anti-obesity action of experimental treatments in this work, particularly those including MET. Induction of energy expenditure based on UCP1-independent fatty acid oxidation in white fat may ameliorate adiposity [51]. Interestingly, even if there were no signs of WAT browning or *Ucp1* induction (actually *Ucp1* expression was repressed), gene expression results suggested increased capacities for lipolysis and subsequent oxidation of the released fatty acids in iWAT of MET and, especially BC+MET mice, as compared to the untreated HF and, especially, the BC mice (see results of *Lipe*, *Pnpla*2, *Mfn2*, *Pparagc1a*, *Cpt1a* and *Cd36* in Figure 5a). Likewise, observed gene expression changes are suggestive of decreased adipogenesis/lipogenesis (downregulation of *Pparg* and *Slc2a4*) and increased fatty acid oxidation (upregulation of *Ppara* and *Cpt1b*) in the visceral rWAT depot of MET and BC+MET mice as compared to the other HFD-fed groups. Unlike in our previous study [12], in this work, we did not observe downregulation of *Pparg* expression in WAT depots following treatment with BC alone, yet there are multiple differences between the two studies, including not only dosage, formulation and duration of the BC treatment, but, most importantly the type of diet, which was a normal-fat diet in the previous study and a high-fat diet in the present one.

Decreased total liver lipid content in the groups treated with BC might agree with a previous report of a protective effect of BC (mainly 9-cis, from algal origin) against the development of hepatic steatosis in LDL receptor knockout mice fed an HFD [52]. BC-derived metabolites might be involved since exogenous retinoic acid treatment in mice decreases triglyceride content and enhances fatty acid catabolism in the liver [17]. A recent review concluded that there is significant evidence from human studies suggesting a link between high BC consumption and circulating levels and lower incidence of non-alcoholic fatty liver disease, yet further studies in ad-hoc animal models are required to probe the benefits of dietary BC supplementation against fatty liver [53]. Gene expression results were suggestive of enhanced hepatic fatty acid catabolism (upregulated *Ppara* and *Ppargc1a*), enhanced autophagy and, hence, possible lipophagy (upregulated *Atg7*, *Becn1* and *Foxo3a*) and decreased de novo lipogenesis (downregulated *Gck*) in the BC+MET group compared with the other HFD-fed groups. However, except for *Atg7*, gene expression differences failed to reach statistical significance, and, moreover, changes in these genes could not explain decreased lipid content in the BC group.

We found dramatic effects of MET treatment increasing skeletal muscle capacities for substrate (glucose and fatty acid) uptake, mitochondrial fatty acid oxidation and insulin sensitivity that may contribute to metabolic benefits. These results are in keeping with previous studies indicating that MET administration increases glucose disposal [54], fatty acid oxidation [55,56] and insulin sensitivity [57] in skeletal muscle. To be noted, the magnitude of gene expression changes elicited by MET treatment was much higher in skeletal muscle than in the liver. The effects of BC treatment on gene expression in skeletal muscle were milder than effects of MET treatment and included both potentially beneficial changes (e.g., upregulation of *Insr, Irs1* and, to a lesser extent, *Lpl*, *Cd36*, *Cpt1b*, *Mcad* and *Ucp3*) and harmful changes (e.g., downregulation of *Ppargc1a* and *Slc2a4*) relative to expression levels in the HF control group. Downregulation of muscle *Ppargc1a* and *Slc2a4* expression in the BC group was counteracted in the BC+MET group, whereas observed gene inductions by single BC and MET treatments were not additive in the cotreatment group. Interestingly, compared to the other groups, the MET and BC+MET groups displayed mRNA levels of GLUT4 higher in skeletal muscle and BAT tissues and lower in visceral (retroperitoneal) fat tissue, which suggest increased partitioning of postprandial glucose to substrate consuming/thermogenic tissues in these groups.

Considering that rodents are very efficient BC cleavers [58,59] and the relatively low BC dose used, it is to be expected in this work that all the BC administered is converted to vitamin A retinoids in the enterocytes, and from them distributed to tissues. Observed effects in groups receiving BC may therefore rely, directly or indirectly, on local changes in retinoid levels. Indeed, as discussed in previous paragraphs, some of the changes observed in these groups are in good concordance with changes known to be elicited in mice after retinoic acid treatment. However, as a limitation of this study, levels of BC, retinoids and MET in tissues were not analyzed, and therefore eventual correlations of observed changes with tissue levels of these compounds were not evaluated.

Regarding the translation of the pre-clinical results in this work to humans, although no direct translation can be performed, it is interesting to note that unpublished data from the MARK-AGE Project—a large European cross-sectional study on biomarkers of aging [60]—showed significantly better blood parameters related to glucose control (lower glucose and insulin) and lipid metabolism (higher HDL, lower triglycerides) as well as a trend concerning systemic inflammation (lower c-reactive protein) in participants taking metformin with high plasma BC levels compared to those taking metformin with low plasma BC levels (personal communication by Daniela Weber and Alexander Bürkle on behalf of the MARK-AGE Project). Furthermore, the combination of metformin and a carotenoid supplement rich in BC was used in the treatment of obesity in children and adolescents with satisfactory results (personal communication by Jose A. Canas).

Altogether, results herein support the benefits of relatively low dose BC and MET treatments against the development of HFD-induced obesity and its metabolic burden and specific benefits of the BC+MET cotreatment. Some of the observed effects of cotreatment were basically attributable to the BC component (e.g., positive effects on blood glucose, systemic insulin sensitivity and BAT appearance; decreased liver lipid content) or the MET component (e.g., most gene expression changes in skeletal muscle). Other interesting responses were elicited by both the BC and MET individual treatments and were present in the cotreatment group though without an additive effect (e.g., weight loss upon a 6 h-fast). Among the specific benefits of the cotreatment, a clearer effect opposing body weight gain and hyperglycemia and, especially, its distinct effect opposing subcutaneous adipocyte hypertrophy on an HFD are to be highlighted. Clearly, further mechanistic studies are required to understand the biological bases of the complex and varied effects unveiled in this study. In any case, it is interesting to note that, with few exceptions (such as the effect on skeletal muscle Irs1 expression), on an HFD, the BC+MET cotreatment elicited “all the good” of the individual BC and MET treatments, compensated “the bad” and generated additional favorable effects.

## Data Availability

Not applicable.
